# Dexmedetomidine versus Midazolam in Procedural Sedation. A Systematic Review of Efficacy and Safety

**DOI:** 10.1371/journal.pone.0169525

**Published:** 2017-01-20

**Authors:** Clemens R. M. Barends, Anthony Absalom, Baucke van Minnen, Arjan Vissink, Anita Visser

**Affiliations:** 1 Department of Anesthesiology, University of Groningen and University Medical Center Groningen, Groningen, The Netherlands; 2 Department of oral and maxillofacial surgery, University of Groningen and University Medical Center Groningen, Groningen, The Netherlands; Imperial College London, UNITED KINGDOM

## Abstract

**Objectives:**

To systematically review the literature comparing the efficacy and safety of dexmedetomidine and midazolam when used for procedural sedation.

**Materials and Methods:**

We searched MEDLINE, EMBASE and COCHRANE for clinical trials comparing dexmedetomidine and midazolam for procedural sedation up to June 20, 2016. Inclusion criteria: clinical trial, human subjects, adult subjects (≥18 years), article written in English, German, French or Dutch, use of study medication for conscious sedation and at least one group receiving dexmedetomidine and one group receiving midazolam. Exclusion criteria: patients in intensive care, pediatric subjects and per protocol use of additional sedative medication other than rescue medication. Outcome measures for efficacy comparison were patient and clinician satisfaction scores and pain scores; outcome measures for safety comparison were hypotension, hypoxia, and circulatory and respiratory complications.

**Results:**

We identified 89 papers, of which 12 satisfied the inclusion and exclusion criteria; 883 patients were included in these studies. Dexmedetomidine was associated with higher patient and operator satisfaction than midazolam. Patients receiving dexmedetomidine experienced less pain and had lower analgesic requirements. Respiratory and hemodynamic safety were similar.

**Conclusions:**

Dexmedetomidine is a promising alternative to midazolam for use in procedural sedation. Dexmedetomidine provides more comfort during the procedure for the patient and clinician. If carefully titrated, the safety profiles are similar.

## Introduction

Procedural sedation can provide more comfort for the patient and an easier procedure for the clinician for painful or unpleasant diagnostic or therapeutic procedures. It may be preferred over general anesthesia due to physiological, financial and logistical considerations,.

Midazolam is one of the classic sedatives for procedural sedation. While midazolam is thought to cause minimal hemodynamic effects, it does have the potential to cause loss of airway reflexes, respiratory depression, and even apnea [1]. If an effective, reliable and safe sedative could be used in general practice, this would benefit a wide range of patients, especially those who are frail, anxious, severely phobic or uncooperative.

Dexmedetomidine (an alpha2-adrenergic agonist) is a relatively new drug, which can also be used for procedural sedation. It has sedative and anxiolytic properties and is known for its analgesic potential owing to a reduction of sympathetic tone. Dexmedetomidine induces dose-dependent effects, ranging from minimal to deep sedation. Moreover, except at doses that cause very deep sedation or general anesthesia, the sedation is reversible. The patient can be easily roused to a lucent state, but when left undisturbed will fall back into a state very similar to natural sleep. These are unique properties among the sedative medications in common use. Dexmedetomidine does not impair the respiratory drive per se and seldom causes apnea. However, it has been shown to impair the respiratory responses to hypoxia and hypercapnia [2] and can cause hemodynamic effects such as hypertension, hypotension and bradycardia [1].

Many studies have compared aspects of the safety and efficacy of midazolam and dexmedetomidine, but the results have not yet been systematically reviewed. Therefore, the aim of our systematic review was to systematically review the current literature on the relative efficacy and safety of dexmedetomidine and midazolam when used as monosedatives for conscious, procedural sedation. We included studies of all types of surgical or diagnostic procedures.

The objective of this systematic review was to answer the following research question: does dexmedetomidine result in more efficacious and safer sedation compared to midazolam in the periprocedural period for adult patients undergoing procedural sedation?

## Methods

### Literature Search

We searched the Cochrane, Pubmed and Embase databases to identify adult human clinical trials comparing the sedative efficacy and/or safety of dexmedetomidine versus midazolam. Studies that compared both drugs without routine use of other sedative medications—other than for rescue from inadequate sedation or analgesia—were eligible for inclusion.

For the Pubmed search (last accessed on June 20, 2016) we used the following search strategy: (("dexmedetomidine"[MeSH Terms] OR "dexmedetomidine"[All Fields]) AND ("midazolam"[MeSH Terms] OR "midazolam"[All Fields]) AND sedation[All Fields]) AND "humans"[MeSH Terms]). The Embase database was searched with a comparable search strategy as used for Pubmed (last accessed June 20, 2016): ('dexmedetomidine'/exp OR dexmedetomidine AND ('midazolam'/exp OR midazolam) AND ('conscious sedation'/exp OR 'conscious sedation')). The Cochrane library was searched for relevant reviews using the following search terms: dexmedetomidine AND midazolam AND sedation. There was no limit on the years considered.

**Fig 1:**
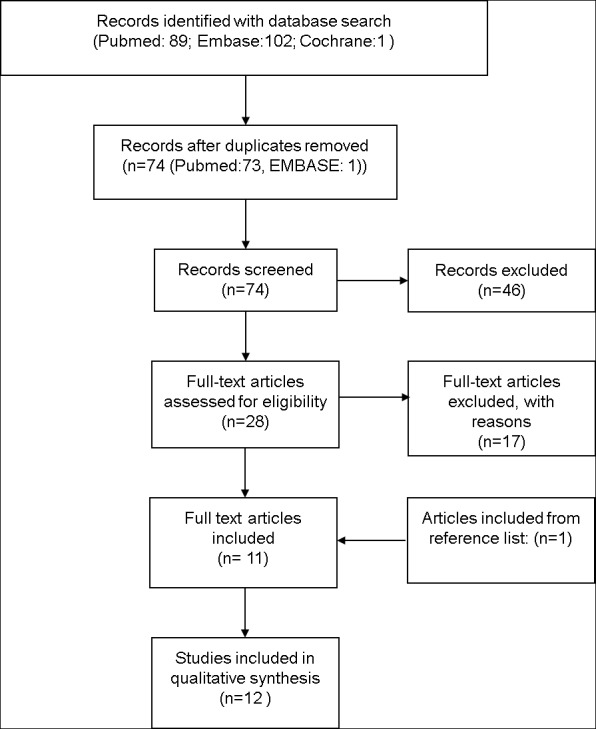
PRISMA Diagram.

The papers thus identified were screened by two authors (CB, AV) by title and abstract for eligibility for inclusion (Fig 1). The resulting papers were read in full (by CB and AV) and the reference lists were scanned for additional material.

Data extraction and assessment of risk of bias was done by the same two authors (CB, AV). Pilot forms were used to extract unfiltered data on all described outcome measures related to efficacy and safety. The outcome measures for the review were selected by means of team discussion (CB, AV, AA, BM). The selected outcome measures were placed onto forms for collecting data from the studies reviewed.

### Study selection

Study selection was based on the following criteria:

Participants: adults receiving procedural sedation

Type of intervention: dexmedetomidine use for procedural sedation

Type of comparison: midazolam use for procedural sedation

Study types: randomized controlled clinical trials

We excluded studies where dexmedetomidine or midazolam was given as part of intensive or critical care and any studies including children. Also excluded were studies where additional medication with sedative properties was given other than as rescue medication.

The Jadad score [3] was used to assess the quality of trials. Disagreements on Jadad scoring were solved primarily by discussion and secondarily by consultation with a third author (AA). We planned to discuss any information from low quality studies (Jadad score < 3) with all authors before using it for conclusions for this review. Statistical data from low quality studies was not included in our statistical analyses.

Patient and operator satisfaction scores and pain scores were used to compare efficacy. If these outcome measures were not expressed numerically in scores or ratings, we used the verbal descriptions provided in the studies.

To compare the safety of dexmedetomidine and midazolam we recorded all reports of hypotension and hypoxia. When no explicit mention of these events was present, reports of hemodynamic and respiratory complications were discussed by three authors (CB, AV, AA) to evaluate their eligibility for inclusion in the results. If the absence of complications was explicitly mentioned or if it was explicitly stated that there was no need for intervention, the incidence of hypotension or hypoxia was assumed to be zero.

### Statistical analysis

To compare the incidences of hypotension and respiratory adverse events, we isolated the studies and patient groups in which patients received either dexmedetomidine or midazolam for subgroup analysis. These pooled incidences of hypotension and respiratory adverse events were compared with the Chi-square test.

## Results

The results of our search strategy according to the PRISMA method [4] are summarized in Fig 1 (Fig 1). We identified a total of 89 papers, of which 12 satisfied the inclusion and exclusion criteria. Because not all studies reported all of the predefined outcomes, our conclusions were based on subgroups of studies reporting the relevant outcomes.

### Description of studies

Twelve publications were direct comparisons of dexmedetomidine with midazolam (Fig 1, Table 1). The included studies were heterogenous with respect to dosages, administration regimens and scoring systems. In 2 of the 12 publications the subjects were human volunteers, not patients. One study investigated hemodynamic changes during sedation with dexmedetomidine or midazolam (or propofol) [5], the other evaluated the effects of these sedatives on the perception of various painful stimuli [6]. A priori power analysis was mentioned in 4 of the 12 studies.

**Table 1:** Study design.

Author	Study comparison	Power analysis	JADAD score	n	ASA	Procedure	Dexmedetomidine dose	Midazolam dose	Use of LA
Alhashemi et al. [7]	DEX versus MDZ	Yes	4	44	1–3	Cataract	1μg/kg load. 0.1–0.7μ/kg/hr IV	20μ/kg stat IV. 0.5mg IV prn	Yes
Apan et al. [8]	DEX versus MDZ	No	5	90	1–3	Cataract	0.25μg/kg/hr IV	25μg/kg/hr	Yes
Cheung et al. [9]	DEX versus MDZ	Yes	5	60	1–2	Oral surgery	1.0μg/kg IV	5mg IV	Yes
Demiraran et al. [10]	DEX versus MDZ	No	3	50	1–2	Endoscopy	1μg/kg load. IV, 0.2μg/kg/hr maint.	0.07mg/kg IV	Yes
Fan et al. [11]	DEX versus MDZ	No	3	60	1–2	Oral surgery	0.1μg/kg/min IV load. 0.2μg/kg/hr IV maint.	0.005mg/kg/min IV load. 0.01mg/kg/hr IV maint.	Yes
Frölich et al. [6]	DEX versus MDZ	Yes	3	86	1	Pain stimuli	0.1–0.8ng/ml IV	10-80ng/ml IV	No
Frölich et al. 2011[5]	DEX versus MDZ	No	2	60	1	None	0.1–0.2–0.4–0.8 ng/ml IV	1-20-40-80 ng/ml IV	No
Hashiguchi et al. [12]	DEX versus MDZ	No	2	40	?	Endoscopy	6.0μg/kg/hr IV load. 0.6μg/kg maint.	0.05mg/kg	Yes
Kaya et al. [13]	DEX versus MDZ	No	5	75	1–2	TURP	0.5μg/kg IV	0.05mg/kg IV	Yes
Liao et al. [14]	DEX versus MDZ	Yes	3	198	1	bronchoscopy	1μg/kg load. 0.5μg/kg/hr maint.	2mg IV stat, 1mg IV prn	Yes
Muttu et al. [15]	DEX versus MDZ	No	1	40	?	Cataract	1μg/kg IV load. 0.05–0.7μg/kg/hr IV	50μg/kg IV load. 2.5–35μg/kg/hr IV maint.	Yes
Ustun et al. [16]	DEX versus MDZ	No	4	20	1	Oral surgery	4μg/kg/h IV	0.4mg/kg/h IV	Yes

LA: Local Anesthetic; DEX: dexmedetomidine; MDZ:mMidazolam; IV: intravenous; IN: intranasal; prn: pro re nata (as needed); stat: statim (once); TURP: Transurethral Resection of the Prostate; AFOI: Awake Fibreoptic Intubation.; load.: loading dose; maint.: maintenance dose.

Of the 12 included studies, 4 were given a final JADAD score of 2 or lower [5,6,12,15] (Table 2). Although all 12 studies were randomized, not all were double-blind trials. Two were volunteer studies in which the authors did not use blinding as this was deemed not feasible or not appropriate [5,6]. The three other studies without double-blinding were from Demiraran et al, Hashiguchi et al. and Liao et al. [10,12,14]. In the first study the patients and the observers were blinded, but not the physician performing the procedure. In the latter two there was no blinding (Table 2).

**Table 2:** Jadad scores for included studies.

	Study described as random ized	Study described as double blind	Description of withdrawals and dropouts	Method of randomisation was described in the paper, and appropriate.	Method of blinding described, and appropriate	Method of randomi-sation described, but inappropriate	Method of blinding describedbut inappropriate	JADADScore
Alhashemi et al. [7]	Yes	Yes	No	Yes	Yes	No	No	4
Apan et al. [8]	Yes	Yes	Yes	Yes	Yes	No	No	5
Cheung et al. [9]	Yes	Yes	Yes	Yes	Yes	No	No	5
Demiraran et al. [10]	Yes	No	Yes	Yes	No	No	No	3
Fan et al. [11]	Yes	Yes	Yes	No	No	No	No	3
Frölich et al. [5]	Yes	No	No	Yes	No	No	No	2
Frölich et al. [6]	Yes	No	No	Yes	No	No	No	2
Hashiguchi et al. [12]	Yes	No	Yes	No	No	No	No	2
Kaya et al. [13]	Yes	Yes	Yes	Yes	Yes	No	No	5
Liao et al. [14]	Yes	No	No	Yes	Yes	No	No	3
Muttu et al. [15]	Yes	No	No	No	No	No	No	1
Ustun et al. [16]	Yes	Yes	Yes	No	Yes	No	No	4

### Efficacy: satisfaction, sedation, analgesia and amnesia

#### Patient satisfaction

Eight studies (n = 597 subjects) measured patient satisfaction by means of numerical rating scales or questionnaires with Likert scales (Table 3). Four of these studies (n = 214) reported dexmedetomidine to be superior in this respect to midazolam [7,8,11,16]. The other four studies (n = 383) [9,10,13,14] did not show a significant difference in patient satisfaction between the drugs (Table 4).

**Table 3:** Study and outcome overview.

	Studies	n
**Hypotension**		
-reporting on incidence of hypotension	10	737
-without significant difference	10	737
-with greater incidence in DEX treated pts	0	0
-with greater effect in MDZ treated pts	0	0
**Respiratory events**		
-reporting on incidences of respiratory events	11	767
-without significant difference	11	797
-with greater incidence in DEX treated pts	0	0
-with greater incidence in MDZ treated pts	0	0
**Efficacy**		
-reporting on patient satisfaction	8	597
-without significant difference	4	214
-with greater pat. satisfaction in DEX treated pts	4	383
-with greater pat. satisfaction in MDZ treated pts	0	0
-reporting on clinician satisfaction	9	637
-without significant difference	5	463
-with greater clinician satisfaction in DEX treated pts	4	174
-with greater clinician satisfaction in MDZ treated pts	0	0
-reporting on analgesia	8	577
-without significant difference	6	443
-with better analgesia scores in DEX treated pts	2	134
-with better analgesia scores in MDZ treated pts	0	0
- reporting on predictability/stability	11	1002
-without significant difference	3	180
-with better predictability in DEX treated pts	8	822
-with better predictability in MDZ treated pts	0	0

DEX: dexmedetomidine; MDZ: midazolam.

**Table 4:** Results for Patient Satisfaction, Clinician Satisfaction, Analgesia and Sedation Predictability/Consistency.

			Patient Satisfaction		Clinician Satisfaction		Analgesia		SPCR
		n	Parameter	Result	Parameter	Result	Parameter	Result	
Alhashemi et al. [7]	DEX versus MDZ	44	VRS/LS	DEX	VRS/LS Scale	DEX	VRS/LS Scale	DEX	MDZ
Apan et al. [8]	DEX versus MDZ	90	BR	DEX	VRS/LS Scale	NS	VASpain	DEX	NR
Cheung et al. [9]	DEX versus MDZ	60	NRS	NS	NRS and VRS	NS	NRS	NS	DEX
Demiraran et al. [10]	DEX versus MDZ	50	VAS	NS	VAS	DEX	VASpain	NS	NR
Fan et al. [11]	DEX versus MDZ	60	VAS	DEX	VRS/LS Scale	DEX	NR		NS
Frölich et al. [5]	DEX versus MDZ	60	NR		NR		NR		NR
Frölich et al. [6]	DEX versus MDZ	86	NR		NR		NR		NR
Hashiguchi et al. [12]	DEX versus MDZ	100	NR		NR		NR		NS
Kaya et al. [13]	DEX versus MDZ	75	BR	NS	VRS/LS Scale	NS	VASpain	NS	NS
Liao et al. [14]	DEX versus MDZ	198	BR	NS	VAS	NS	VASpain	NS	NR
Muttu et al. [15]	DEX versus MDZ	40	NR		VRS/LS Scale	NS	VASpain	NS	DEX
Ustun et al. [16]	DEX versus MDZ	20	VAS	DEX	VRS/LS Scale	DEX	VASpain	NS	NS

SPCR: SedationPredictability/Consistency Rating; VRS/LS:Verbal Rating Score/Likert Scale; BR: Binary rating; VASpain: Visual Analogue Scale for pain; NRS: Numeric Rating Scale; UAA: use of additional analgesia; ISAS: Iowa satisfaction with Anesthesia Score; NR:Not Reported.

#### Analgesia

Eight studies (n = 577) reported on the comparative analgesic effects of dexmedetomidine and midazolam (Table 3). Two of them (n = 134) reported that dexmedetomidine had a greater analgesic effect [7,8]. The other six studies (n = 443) showed no difference in analgesic potency between the drugs [9,10,13–16] (Table 4).

In two studies in which dexmedetomidine treatment resulted in higher patient satisfaction, this treatment was also associated with better analgetic properties compared to midazolam [7,8]. Midazolam has no analgesic effect and can even lower pain thresholds [6]. Likewise, the studies resulting in no preference for dexmedetomidine or midazolam also reported no difference in analgetic potential [9,10,13,14].

#### Clinician satisfaction

Nine studies (n = 637) reported either clinician satisfaction or a measure of ease of performance of the procedure (Table 3). Four of them (n = 174) reported a significant difference in favor of dexmedetomidine in terms of clinician satisfaction with the sedation [7,10,11,16]. In the other five studies (n = 463) dexmedetomidine and midazolam resulted in equal clinician satisfaction [8,9,13–15] (Table 4).

Kaya et al. reported that dexmedetomidine use, when compared to midazolam, was associated with more patients reaching the desired level of sedation [13]. Muttu et al. and Cheung et al. [9,15] mentioned that several patients became restless, aggressive or agitated after midazolam administration. This reaction was not reported in dexmedetomidine-treated patients in any of the included studies.

### Safety

#### Respiratory effects

In 11 studies (n = 767) both the number of patients included and the number of respiratory adverse events or complications was reported [5,7–16]. We found no difference in the incidence of respiratory events in the pooled results from the high quality studies. We found 20 events of hypoxia among 281 dexmedetomidine-treated patients, compared to 24 events among 280 midazolam-treated patients (p = 0.52; Table 5).

**Table 5:** Incidences of hypotension and hypoxia / hemodynamic or respiratory complications.

Author	Study design	Incidence of hypotension/complications DEX	Incidence of hypotension/complications MDZ	Group with greatest incidence of hypotension	Incidence of hypoxia DEX	Incidence of hypoxia MDZ	Group with most respiratory adverse events	SupplO_2_ (l/min)
Alhashemi et al. [7]	DEX versus MDZ	0	0	NS	0	0	NS	2
Apan et al. [8]	DEX versus MDZ	0	0	NS	0	0	NS	2
Cheung et al. [9]	DEX versus MDZ	0	0	NS	6	4	NS	0
Demiraran et al. [10]	DEX versus MDZ	0	0	NS	0	2	MDZ	
Fan et al. [11]	DEX versus MDZ	0	0	NS			NS	0
Frölich et al. [5]	DEX versus MDZ	NR	NR	NR	0	0	NS	0
Frölich et al. [6]	DEX versus MDZ	NR	NR	NR	NR	NR	NR	0
Hashiguchi et al. [12]	DEX versus MDZ	2	2	NS	1	0	NS	0
Kaya et al. [13]	DEX versus MDZ	2	0	NS	0	0	NS	4
Liao et al. [14]	DEX versus MDZ	6	7	NS	14	18	NS	4
Muttu et al. [15]	DEX versus MDZ	0	0	NS	0	0	NS	0
Ustun et al. [16]	DEX versus MDZ	0	0	NS	0	0	NS	0

DEX: dexmedetomidine; MDZ: midazolam; PBO: placebo; NS: no significant difference in effect; NR: not reported. SpO_2_:arterial oxygen saturation (measured by pulse oxymetry).

#### Hemodynamic effects

Except for two studies [5,6], all studies reported the incidence of hemodynamic adverse events. Among ten studies (n = 737) the incidence was 0% for both groups in seven studies [7–11,15,16], while in three studies the number of adverse events was very low and the difference between groups was not statistically significant [12–14] (Table 5).

The data from the eight good quality studies was pooled, showing a similar incidence of hypotension: 10 events of hypotension among 281 dexmedetomidine-treated patients compared to 7 events among 280 midazolam-treated patients (p = 0.80) [7–11,13,14,16] (Table 5).

Finally, two studies [12,14] reported that hypertension occurred as an unwanted reaction in midazolam-treated patients, whereas the hypertensive response to stress was attenuated in the dexmedetomidine-treated patients.

## Discussion

For adult patients undergoing procedural sedation, dexmedetomidine results in more efficacious sedation than midazolam in the periprocedural period. The safety profile of both drugs appears to be similar. We found that dexmedetomidine has potential benefits over midazolam when used for procedural sedation. No studies reported that patients or clinicians were more satisfied with the result of midazolam sedation, whereas several studies found dexmedetomidine use to be associated with greater patient and clinician satisfaction and greater analgetic potential. The safety of both drugs seems to be similar with respect to respiratory or hemodynamic complications.

### Patient and clinician satisfaction

Patients expect procedural sedation to provide them with comfort during an otherwise stressful or painful period. Alleviation of pain and discomfort is an important determinant for patient satisfaction scores. This may explain the higher patient satisfaction scores for dexmedetomidine in several studies [7,8]. Dexmedetomidine has intrinsic analgetic potential, whereas midazolam can actually increase pain perception [6]. Only Ustun et al. reported greater patient satisfaction without better analgesia for dexmedetomidine [16]. This was a crossover study in which patients were treated sequentially with both sedatives.

All studies, except for the volunteer studies of Frölich et al. [5,6], used additional local anesthesia. This is in accordance with the generally accepted view that sedatives for conscious sedation should never be used without additional analgesia.

Midazolam also provides amnesia. Although this may be preferred by some patients, for instance those with dentophobia, amnesia could also be considered undesirable for this group of patients. These patients aim to alleviate their fear through gradual exposure, and amnesia prevents this learning effect.

Like patients, clinicians in the selected studies tended to have a preference for dexmedetomidine. Based on their additional comments in these studies, this preference might be explained by the improved cooperation of patients treated with dexmedetomidine and the absence of paradoxical reactions in this group [9,11,15]. The better cooperation of patients treated with dexmedetomidine is due to the unique properties of dexmedetomidine sedation. The patients can be woken to a lucid state from what seems like natural sleep, to follow instructions and return to a sedated state when left undisturbed.

Paradoxical reactions are a rare but well known side effect of benzodiazepines. The occurrence of restlessness, agitation and even aggression is very disturbing to the patient as well as to the clinician. The etiology is unclear but children and the elderly are more prone. Although the reported incidence is <1% these reactions can prevent clinicians from completing the procedure. Moreover, the outright hostility seen in some patients exhibiting a paradoxical reaction to midazolam presents a danger to both patient and clinician [17]. Dexmedetomidine is not known to cause these reactions and no such reactions were reported in dexmedetomidine-treated patients in the included studies.

All of the above findings can be explained from the pharmacodynamics of both drugs. Midazolam produces its effects through the GABAa receptors and inhibits the excitatory reaction of the brain to stimuli [1]. Dexmedetomidine does not produce such central cerebral inhibition; it affects the locus coeruleus. This is a central neural pathway playing a key role in inducing natural sleep. Dexmedetomidine has been shown to have the ability to improve natural sleep when given to intensive care patients in both low, non-sedative dosages [18] and in sedative dosages [19]. Dexmedetomidine also lowers sympathetic tone. It’s mechanism of action lowers fear and anxiety, whereas midazolam inhibits a reaction of the patient to uninhibited stimuli. This may explain why sedation with dexmedetomidine is preferred by many patients over midazolam, which is in line with the crossover study of Ustun et al. [16].

### Safety

#### Respiration

In the pooled results dexmedetomidine and midazolam did not differ in terms of respiratory safety. This was surprising because midazolam is well known for causing respiratory depression [1], and we expected that dexmedetomidine treatment would result in fewer cases of hypoxia. Most of the included studies had careful infusion protocols for both drugs. And although midazolam has a rapid onset time of 2 to 3 minutes, the effect site concentration peaks only after approximately 13 minutes [20,21]. Repeat boluses may be given too early, which can lead to overdosing and hypoxia.

#### Hypotension

Dexmedetomidine has a reputation of causing hypotension, which is sometimes preceded paradoxically by hypertension. In contrast, midazolam is known for its hemodynamic stability. However, the hypotensive effect of dexmedetomidine can be mitigated by preventing rapid infusion and by not using bolus dosing. High peak plasma levels are responsible for the complex hemodynamic effects of dexmedetomidine [22]. In all studies the loading dose of dexmedetomidine was infused slowly. Alternatively, intranasal administration of dexmedetomidine avoids high peak plasma levels but still results in adequate plasma levels after uptake, as shown by Iirola et al. [23]. Moreover, the usefulness of intranasal administration for procedural sedation has been demonstrated by Zhang et al. and Nooh et al. [24,25].

Careful dosing, preferably by titration, is the key to procedural sedation. Within the confines of carefully protocollized studies, dexmedetomidine (when used with slow loading dosages) and midazolam (in a careful infusion regimen) would appear to have similar safety profiles. For general practice, this would require intravenous access and infusion pumps for titration, and appropriate monitoring would still be needed. When patients are sedated with dexmedetomidine, they remain rousable. However, it should be remembered that like midazolam, dexmedetomidine has moderately slow pharmacokinetics, so that during the recovery period, the patient may be sleepy when not stimulated. As with midazolam, close observation is necessary for a period of time before discharge commensurate with the pharmacokinetics of the agent. Dexmedetomidine, however, seems to yield better results with respect to patient and clinician satisfaction. And the possibility of using this drug safely with intranasal administration may be very useful for office-based procedural sedation. The safe use of dexmedetomidine in the general population, and more specifically the frail or the elderly should be the subject of further investigation before this treatment can be used in the office-based or nursing home-based care setting.

#### Limitations

The included studies varied widely with respect to dosing regimens, procedures and outcome measures. Also, not all studies reported in enough detail on these outcome measures. This prevented us from performing more formal meta-analyses.

We excluded studies where analgesics with an additional sedative effect were given other than as rescue medication and placebo-controlled studies where midazolam served as rescue medication. This led to the exclusion of many papers studying dexmedetomidine versus midazolam for conscious sedation. However, the exclusion of these studies allowed for a more precise comparison of the effects of both drugs without accounting for numerous (unpredictable) pharmacological interactions.

We included four studies of moderate to low quality[5,6,12,15]. Frölichs studies [5,6] do compare both drugs but not for procedural sedation.The conclusions of none of these studies conflict the information from this review. Therefore the effect their results have on our conclusions is minimal and non-conflicting. We isolated their results from statistical analysis. Re-calculation without this isolation did not change the outcome for the subgroup analyses.

Midazolam causes profound amnesia. The amnesic effect appears at lower dosages and is more apparent than the sedative effect. This amnesia could have confounded results when patient questionnaires are used after midazolam treatment.

The frail and elderly are an underrepresented group in these studies. With only one study including some ASA 3 patients or a substantial number of patients aged over 60, the safety and efficacy of dexmedetomidine compared to midazolam for use in procedural sedation in frail patients has not been well assessed [8]. Su et al. [26] have shown in a recent publication how the use of low-dose dexmedetomidine for sedation in the elderly in the ICU is safe and has a preventive effect on the development of delirium after surgery. The occurrence of delirium is of concern in the treatment of elderly people and the possibility to prevent it from occurring may prove to be another benefit of the use of dexmedetomidine in procedural sedation.

## Conclusion

We have shown that dexmedetomidine has advantages over midazolam in terms of reliability, analgesia and patients’ and clinicians’ satisfaction. Moreover, within the scope of this review, dexmedetomidine and midazolam appear to have a similar cardio-respiratory safety profile when both are carefully titrated. Combined with the use of local anesthesia, dexmedetomidine provides a good alternative for midazolam for procedural sedation.

## Supporting Information

S1 PRISMA Checklist PRISMA Checklist. (DOC)
